# Human-De Brazza’s monkey conflict in Kafa Biosphere Reserve, Kafa Zone South West, Ethiopia

**DOI:** 10.1186/s40850-024-00210-2

**Published:** 2024-08-12

**Authors:** Melaku Haile, Tsegaye Gadisa, Tariku Mekonnen Gutema

**Affiliations:** 1 Bonga University, PhD Candidate in Jimma, University, Jimma, Ethiopia; 2 Tsegaye Gadisa Associate Professor in Department of Biology, Collage of Natural Science Jimma University, Jimma, Ethiopia; 3 Tariku Mekonnen Gutema Associate Professor in Department of Natural Resources Management, Jimma University, Jimma, Ethiopia

**Keywords:** *De Brazza’s monkey*, *Kafa Biosphere Reserve*, *Crop raiding*, *Human- De Brazza’s* monkey conflict, Conservation

## Abstract

**Background:**

Human-wildlife conflict (HWC) is any interaction between humans and wildlife that arises when wildlife necessities encroach on those of the human population. It affects all areas where animal and peoples cohabit regardless of geography or climatic circumstances; but the burden is great in developing nations. De Brazza’s monkey (DM) (*Cercopithecus neglectus*) is one of the most unusual species in the group of Old-World monkeys commonly known as guenons. The De Brazza’s monkey is distributed in different parts of African forests from Guinea to Ethiopia. This study was conducted in Kafa Biosphere Reserve, Kafa Zone, South West Ethiopia, to assess the causes of human wildlife conflict in the area. The methods used were, household questionnaire, focus group discussion and direct field observation from June 2022 to May 2023.

**Results:**

The study revealed that the major causes of human De Brazza’s monkey conflicts were; habitat destruction 52.9%, (*n* = 72), human proximity to natural forest, 25.7%, (*n* = 35) and increasing of its population size 8.1 %, (*n* = 11). These monkeys’ raids crop usually early in the morning 42.6%, (*n* = 58), and late evening 29.4%, (*n* = 40). Maize was the most damaged crop by De Brazza’s monkey followed by coffee. The study also confirmed that guarding was the most common method used to protect crops from crop raiding wildlife in the area. Majority 66.2%, (*n*= 90) of the informants had negative attitude but 22.1%, (*n* = 30) had positive attitude towards De Brazza’s monkey conservation.

**Conclusion:**

The study discovered that, in contrast to olive baboons and grivet monkeys in the area, De Brazza's monkeys were not previously identified as crop raiders; however, they are currently causing damage to crops, especially maize and coffee crops. This could be due to habitat destruction and human proximity to the forest boundary. Thus, the conflict between humans and De Brazza's monkeys is escalating. As such, we recommended more research on the population status of the monkeys and strategies for coexist in the area.

**Supplementary Information:**

The online version contains supplementary material available at 10.1186/s40850-024-00210-2.

## Background

Human-wildlife conflict (HWC) is any kind of interaction that arises when the needs of humans and wildlife overlap and results in negative costs to both humans and wild animals [1]. It occurs in all areas where wildlife species and humans live in close proximity and share resources, regardless of geography or climatic conditions [2]. But the burden is greatest in developing nations, where the majority of the populations live in rural areas and rely on subsistence agriculture and livestock husbandry for their livelihood [3].

The conflict between humans and wildlife has occurred since the dawn of humanity [4]. However, severities of the conflict are increasing from time to time due to rapid growth of human population consequently resulting in severe competition for resources in areas previously used by wildlife [5]. Change in land use forms, degradation and destruction of a species habitat, raising interest in ecotourism, an increase in livestock populations and the competitive exclusion of wild herbivores and an increase in wildlife populations has intensified the occurrence of HWC [6].

Crop raiding by wild animals is a major cause for serious conflict between wild animals and the local people especially near protected areas that have sizable population of wildlife species [7]. Many wildlife species come into conflict with humans when they damage crops and this might lead to wildlife mortality as a result of retaliatory actions by humans [8]. Among wildlife species, non-human primates (NHP) are well known in their crop foraging behavior. They are adaptable and opportunistic in feeding different crops which enable them to take advantage of utilizing new resources thus are often in potential conflict with humans [9]. A number of primate species including baboons and chimpanzees are considered the most serious crop raiders because of their intelligence, adaptability, wide dietary range, complex social organization and aggression [10]. De Brazza′s monkey (DM) is also considered agricultural pest in Kenya, where it is observed for raiding maize and potatoes [11].

De Brazza’s monkey (*Cercopithecus neglectus*) is one of the species in the group of Old-World monkeys commonly known as guenons [12]. It is one of the most handsome primate species with unmistakable feature such as white beard and muzzle; highlighted by a chestnut brown patch [13]. Both males and females have grey agouti bodies with black extremities and tail along with a white rump. The distinctive white beard begins under nasal passages and continues down, spreading in their entire mouth and down below their chin. It has extensive cheek pouches used to store food whilst foraging and its tail is none prehensile [14]. The species is fairly common in riparian and swamp forests in the Congo Basin, in southeast Cameroon, Equatorial Guinea, and Angola but it is rare, and found only in isolated pockets, in some parts of extreme east and west Uganda, western Kenya and southwest Ethiopia [15]. The DM is omnivorous and feeds a variety of foods items including fruits, flowers, leaves, berries, and certain invertebrates [15].

Nowadays, managing HWC is becoming very crucial issue in wildlife conservation in areas where the needs of humans and wildlife overlap. Understanding when and why the conflict began, the current trend of the conflict and the distribution of the species is essential for deigning sound management strategies which can benefit both the local people and the wildlife species involved in the conflict. Thus, studies on conservation threats of NHP are important step toward developing effective management plans for conservation. In addition, ecosystems and habitats are alarmingly being dominated by humans, which trigger many species, including NHP, to exploit new human resources to survive [16].

[17] reported the presence of DM in Kafa Biosphere Reserve (KBR). The species is not familiar to most of the residents of the region except farmers who produce crops close to forest edge or others whose livelihood is dependent on forest and forest products. Recently, there are increasing reports of crop damage by DM from local residents that live near to forest edges which might lead to human-De Brazza’s monkey conflict (HDMC). In addition, local communities are likely to develop negative perception towards the conservation of the species because of its crop raiding behavior. This study aimed to provide baseline information on the current situation of HDMC and the perceptions of local residents towards its conservation. The authors of the manuscript predicted that there is no gender difference in the perception of the residents towards the conservation of De Brazza′s monkey.

## Materials and methods

### Description of the study area

**Fig. 1:**
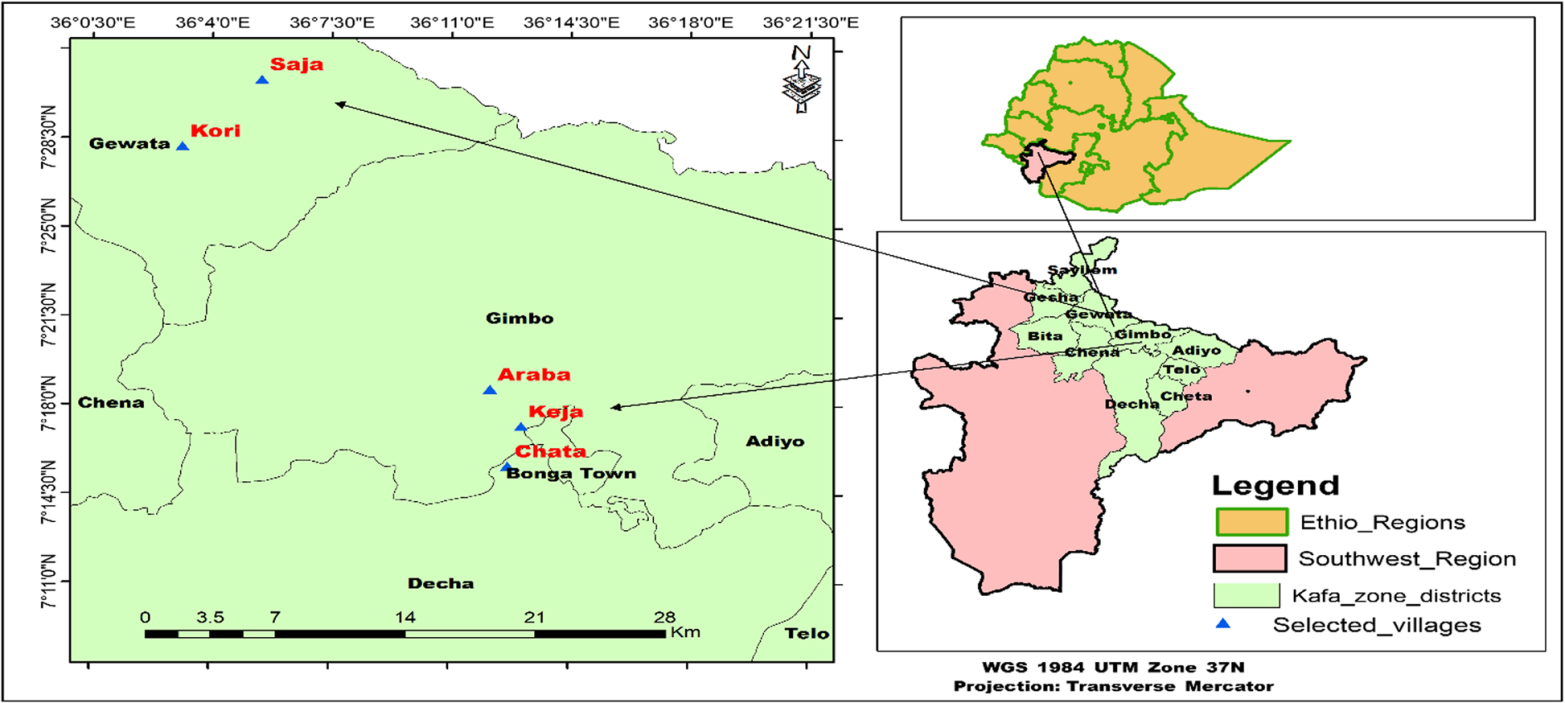
Map of the study area

Kafa Biosphere Reserve is located in Kafa Zone, South West Peoples Regional State (SWPRS), that lies within the latitude of 07°8' to 07°26' North and longitude of 35° 53' to 36^o^ 36' East (Fig. 1). The total land area of the zone is 10,602.7 km^2^_._ The Biosphere Reserve has a total area of 760,000 hectares, of which 420,000 hectares is forest and is included in the catchment basin of the Gojeb, Dincha, and Woshi rivers. It spans 12 adjacent administrative sectors (referred to as "Woredas"), and 4 urban town administrations. The astronomical location of the biosphere is 36°3'22.51" East & 7°22'13.67" North. KBR was accepted as a UNESCO biosphere reserve and added to the World Network of Biosphere Reserves by UNESCO MAB Paris in June 2010 [18]. It is one of the 34 biodiversity hotspots in the world due to its enormous diversity [19]. KBR is home to 250 plant species, 300 mammal species, and 300 different bird species, some of which are only found here. The altitude of area varies from 500 m asl (in the south) to 3,300 m asl in the northeast.

The Kafa Zone is home to a diverse population consisting of various ethnic groups, with the majority being the Kefficho people at 81.04%. Other significant ethnic groups in the area include the Amhara at 5.72%, Bench at 5.5%, Oromo at 2.35%, and other ethnic groups making up about 5.38% of the population [20]. The presence of multiple ethnic groups in the Kafa Zone contributes to its cultural richness and diversity.

The people have indigenous cooperative structures like “Dafo” “Dadoo” and “Idir” with varying number of participants for different activities. “Dafo” is a community level voluntary participation of about 15 persons to assist in agriculture related or house construction activities for one full day. “Dadoo” is similar with “Dafo” except the time duration and number of participants. “Idir” is a social grouping in which members share the grief of those who lost their loved ones and or assist in kind, finance or moral the sick or with other problems [20].

The forest ecosystem makes an important contribution to the livelihoods of people in the area in a variety of ways such as for hanging cylindrical log beehives, provide shade for coffee and a variety of commercially valuable spices, supply rural communities with fuelwood, timber, for household consumption and for sale [21].

The economy of the society is primarily based on subsistence farming, the sale of wild coffee, and the utilization of natural resources such as the forest for various purposes including food, building materials, medicinal plants, animal feed, and honey. The agriculture, the main base of the society’s economy, accounts for 41% of the GDP and 80% of exports and the labor force [22]. Also, the survival farming is prevalent, with local farmers harvesting coffee trees and honey from wild bees for personal use and local markets [23]. Additionally, crops such as False Banana (*Ensete ventricosum)* and Teff (*Eragrostis tef*) are commonly cultivated in the region. The local population has adapted their land use practices, traditions, and customs over centuries to harmonize with nature [23].

Climate, soil, and vegetation is incredibly diverse due to the wide range of landforms. Numerous lush valleys and lowlands, which generally run through the center of the biosphere reserve, connect the mountains and marshes [24]. The Core, Buffer, and Transition zones are the biosphere reserve's three main management focal zones [25].

Data collections were conducted from June 2022 to May 2023 using structured questionnaires, focus group discussion and direct field observation covering the wet and dry seasons. A reconnaissance survey was conducted for two months (April to May 2022) before the beginning of the actual data collection. During the survey period all areas of KBR were assessed and the areas where De Brazza’s monkey present and had conflict with nearby community members were identified.

### Sampling design

Study villages were selected purposively based on the information gathered during the reconnaissance survey that indicated the presence of HDMC and their proximity to the forests of KBR. Accordingly, from the total of four villages in Saja, two villages (Saja and Kori) with total household number102, 88 respectively and from six villages Around Bonga town three villages (Chata, Keja and Araba) with total household population 94, 78, and 92 were selected for this study and their connection with the border of the forest was recognized during the reconnaissance survey. The sample size was determined using [26, 27]; a sampling technique for small populations, hence 30% of the total households (*N* = 454) were taken as a sample population (*n* = 136). Proportional number of households ([28] from Saja, 26 from Kori, 28 from Chata, 23 from Keja and 28 from Araba) were taken using systematic random sampling technique.

### Household survey

A questionnaire survey consisting of both open and close ended questions was used to gather important information, about socio-demographic data, crops grown in the study area, causes of HDMC, damage caused to crops, types of crops mostly damaged by DM, population trends of DM and community’s attitudes and perceptions towards the conservation of DM. Communities attitude was classified as negative, positive and Neutral. The attitude was taken as negative when the responses were complain, criticize, dislike and bad concept towards DM and other non-human primates, and, positive when the responses were good looking, had advantages to consistency of nature, advantage of conserving natural gifts, important to protecting the natural wealth and wise use of natural resource. The attitude was considered as ‘Neutral’, when, neither compline nor appreciation and mostly disregard to reply. Furthermore, the questionnaire was used to collect protective methods used by the community to minimize their crop loss. A structured questionnaire was prepared in English language and translated into Kafi Noonoo; the language mostly used by the local people.

### Focus group discussion

Focus group discussion was conducted to gather valuable and inclusive information on how and when the DM started crop raiding behavior and the most commonly accustomed time at which it visits the crops. The local community’s perception towards DM and the methods used to protect their crops from pest animals and their expectation from the government in safeguarding their properties were included in the discussions. All the selected villages were included and 5-7 individuals from each village were participated at their village by guidance of the mediator. Participants for the group discussion were selected systematically based on; year of residence in the area (at least 10-15 years), traditional leaders, religious leaders and both sexes with age of >20.

### Direct observation /fild observation

Direct assessment was conducted in the selected villages during the study period. It was used to obtain data on; the type of damaged crops, the magnitude of crop lost by DM and mitigation methods used by the farmers of the area.

### Data analysis

SPSS version 26.0 computer software was used to analyze the data. We used descriptive statistic in a form of percentage and frequency to analyze sociodemographic profiles of the respondents. We compared responses of respondents about production of crops, knowledge of DM, causes of HDMCs, impacts and consequences of the conflict, months of severe crop damage and responses about the attitudes respondents towards DM by using a chi-square test. Graphs, tables and figures were used to summarize and present the data.

## Results

### Socio-demographic profile

Among the respondents 75.7% (*n*= 103) were males. Regarding age of the respondents, 40.4% (*n* = 55) were in the age class of 31- 40 years. The majority 95.6 %, (*n* = 130) of respondents were married. Some 29.4%, (*n* = 40) respondents attended their education up to junior (5-8) education level (Table 1). There was significant difference on educational level of respondents among villages (χ^2^ = 31.847; df =12; *p* < 0.05).

**Table 1:** Demographic characteristics of respondents in the study area

Demographic Characteristic	Category	Number	Percent
Sex	Male	103	75.7
	Female	33	24.3
Age	20-30	27	19.9
	31-40	55	40.4
	41-50	37	27.2
	Above 50	17	12.5
Marital status	Married	130	95.6
	Single	0	0
	Divorced	4	2.9
	Widowed	2	1.5
Educational Status	Illiterate	38	27.9
	Primary (1-4)	39	28.7
	Junior (5-8)	40	29.4
	Secondary (9-12)	19	14

Among the respondents, 34.6%, (n= 47) possess 1ha of farmland, 6.6 %, (*n*= 9) own farmland greater than 3ha and the rest possess less than 1ha of farmland. Regarding the length of time they lived in the area, 35.3%, (*n*= 48) of the respondents lived in the area from 10 to 20 years and 24.3% (*n*= 33) of them lived for 10 years while 3.7%, (*n*= 5) of them lived for more than 50 years. The response of respondents in different age groups and different education levels had significant difference on the size of farmland they possess in the study area (χ^2^ = 31.147; df =12; *p* < 0.05) and (χ^2^ = 25.145; df =12; *p* < 0.05) respectively.

More than half 50.4% (*n* = 69) of the respondents earn their income from crop farming and animal rearing while 27.9% (*n* = 38) from crop production, animal rearing and other activities.

Maize was the most cultivated crop in the area 100%, (*n* = 136) followed by coffee 81.6%, (*n* = 111), while the least 28.7 %, (*n* = 39) was fruits. There was significant difference among respondents on production of barley (χ^2^_=_24.070; df = 4; *p* < 0.05), bean (χ^2^_=_12.003; df = 4; *p* < 0.05), sorghum (χ^2^_=_ 9.601; df = 4; *p* < 0.05), teff (χ^2^_=_10.839; df = 4; *p* < 0.05), vegetables (χ^2^ = 10.840; df = 4; *p* < 0.05) and fruits (χ^2^ = 8.296; df = 4; *p* < 0.05) (Table 2).

**Table 2:** Type of crops grown in the area (based on respondents’ response and field observation)

Village	Type of crops									
	maize	barley	bean	pea	haricot bean	sorghum	teff	vegetables	fruits	coffee
Saja	31(100%)	23(16.9%)	15(11%)	16(11.7%)	20(14.7%)	16(11.7%)	22(16.1%)	12(8.8%)	6(4.4%)	27(19.8%)
Kori	26(100%)	18(13.2%)	14(10.3%)	15(11%)	17(12.5%)	18(13.2%)	18(13.2%)	10(7.3%)	4(2.9%)	21(15.9%)
Chata	28(100%	8(5.8%)	13(9.5%)	12 (8.8%)	21(15.4%)	16(11.7%)	12(8.8%)	16(11.7%)	13(9.5)	24(17.6%)
Keja	23(100%)	5(3.6%))	3(2.2%)	3(2.2%)	12(8.8%)	6(4.4%)	8(5.8%)	17(12.5%)	8(5.8%)	15(11. %)
Araba	28(100%)	16(11.7%)	8(5.8%)	17(12.5%)	20(14.7%)	14(10.3%)	16(11.7%))	10(7.3%)	8(5.8%	24(17.6%)
Total	136 (100%)	70(51.5%)	53(39%)	63(46.3%)	90 (66.2%)	70(51.5%)	76(55.9%)	65(47.8%)	39(28.7%)	111(81.6%)

Among domestic animals, chicken was reared by 61.0 %, (*n* = 83) of the respondents followed by sheep 55.1%, (*n*= 75) and equines were reared by 35.3, % (*n* = 48) respondents.

### Human – De Brazza’s monkey conflict

Most respondents 90.4%, (*n* = 123) stated that crop damage was common in their village, with non - human primates (NHP) being the primary cause 73.5%, (*n* = 100). This study confirmed that DM is one of the NHP in the study area, currently damaging certain crops (maize and coffee), and most of the communities know the species. The majority of respondents who had farms on the forest's edge 90.4%, (*n* = 123) were familiar with DM, but community members who lived far from the forest edge and whose livelihood was not linked to forest products 5.1%, (*n* = 7) were less familiar with it. However, the response of respondents in different villages had significant difference regarding the knowledge of DM (χ^2^ =10.611, df = 4, *p* < 0.05).

Most respondents 90.4%, (*n* = 123) replied that they had conflict with DM and some 9.6%, (*n* = 13) have no conflict with it. Of the total respondents74.3% (*n* = 101) mentioned that DM began crop raiding in the last 10 -20 years, but some respondents 16.2%, (*n* = 22) reported that it started recently (less than 10 years).

**Fig. 2:**
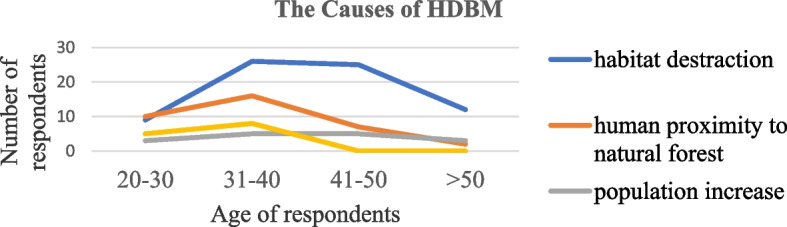
Response of respondents in different age groups about the causes of HDMC in villages of the study area

More than half 52.9% (*n* = 72) of the respondents claimed habitat destruction as the main cause of HDMC followed by human proximity to natural forest 25.3%, (*n* = 35). There was significant difference on causes of HDMC among different age group in the area (χ^2^ = 18.128; df = 9; *p* < 0.05) (Fig. 2).

**Fig. 3:**
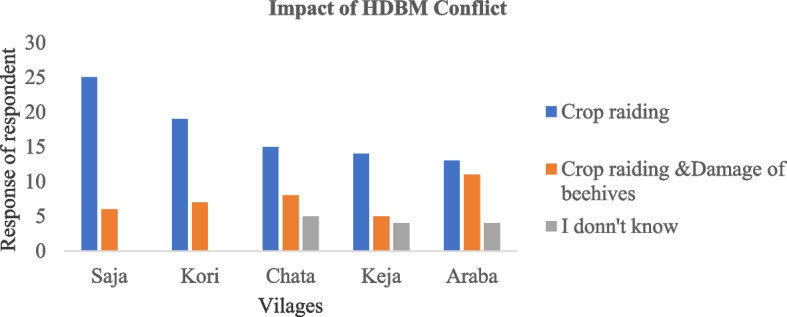
The response of respondents on impacts of HDMC at the study villages

Concerning the impact of HDMC, 63.2%, (*n* = 86) of the respondents reported yield loss by crop raiding as the main impact of HDMC, followed by crop raiding and damage of beehives 27.2%, (*n* = 37). There was significant difference in the response of respondents on impact of HDMC among villages of the study area (χ^2^ =15.642; df = 8; *p* < 0.05) (Fig. 3)

**Fig. 4:**
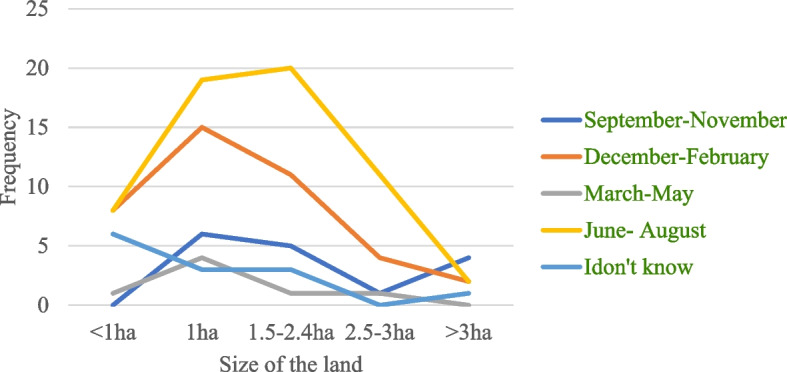
The response of respondents with different land size on months of severe crop damage

Regarding the time of crop raiding by DM, 42.6% (*n* = 58) of respondents replied that DM usually raids crops early in the morning (6:00-7:00AM) while 29.4%, (*n* = 40) of them said it raid crops late in evening (11:30-12:30 PM). However, it raids coffee at any time of the day. Regarding severity of crop damage by DM, 41.1% (*n* = 60) of the respondents replied that it causes severe crop damage from June–August, while 29.7% (*n* = 40) of them reported severe crop damage occurs from December - February. There was significant difference in response of respondents with different land size on the months of severe crop damage (χ^2^ = 26.674; df =16; *p* < 0.05) (Fig. 4).

Some (44.1%, n = 60) of respondents replied that DM raids crop more in summer followed by 29.4%, (n = 40) in winter (Table 3).

**Table 3:** Response of respondents on seasonal damage of crops in the study area.

Seasons	Frequency	Percent
Autumn	16	11.8
Winter	40	29.4
Spring	7	5.1
Summer	60	44.1
I don’t know	13	9.6
Total	136	100

Most respondents 90.4%, (*n* = 123), reported maize as the most damaged crop preceding coffee, which was 77.9%, (*n* = 106) (Table 4) There were significant differences in the response of respondents on damage of maize and barley among villages of the study area (χ^2^ =10.611; df = 4; *p* < 0.05), (χ^2^=31.605; df = 4; *p* < 0.05) respectively.

**Table 4:** Response of respondents about crops raided by DM in the study area

Villages	Does DM raid___?											
	Maize		Barley		Haricot bean		Vegetables		Fruits		Coffee	
	Yes	No	Yes	No	Yes	No	Yes	No	Yes	No	Yes	No
Saja	100%	0%	77.40%	22.60%	25.80%	74.20%	48.40%	51.60%	51.60%	48.40%	87.10%	12.90%
Kori	100%	0%	80.70%	19.20%	26.90%	73.10%	42.30%	57.60%	46.20%	53.80%	80.80%	19.20%
Chata	82.10%	17.80%	35.10%	64.30%	21.40%	78.26	46.40%	53.60%	46.40%	53.60%	71.40%	28.60%
Keja	28.60%	17.40%	17.40%	82.60%	21.70%	78.30%	52.20%	47.80%	60.80%	39.10%	69.60%	30.40%
Araba	85.70%	14.30%	42.80%	57.10%	39.30%	60.70%	39.30%	60.70%	64.30%	35.70%	78.60%	21.40%
Total	90.40%	9.50%	52.20%	47.80%	27.20%	72.80%	45.60%	54.40%	53.70%	46.30%	77.90%	22.10%

**Fig. 5:**
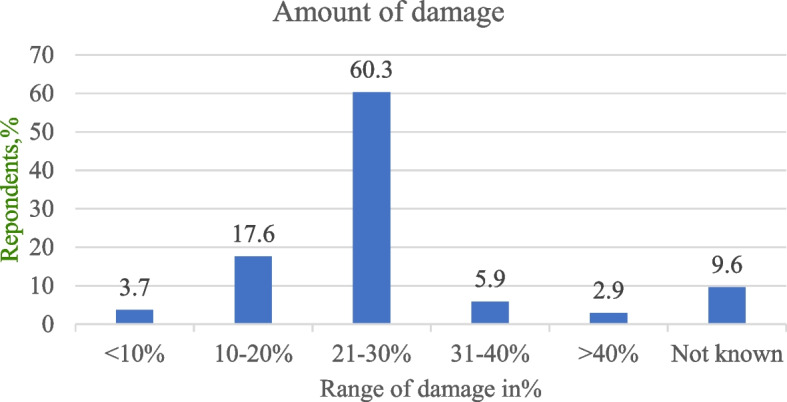
Response of respondents about the amount of crop damage in the study area

Concerning the amount of crop yield reduction by DM, 60.3% (*n* = 82) of respondents reported that they lose 21-30 % of their crop yield in a single cropping season by this primate, while 2.9 %, (*n* = 4) of them claimed that the loss could be greater than 40% (Fig. 5).

**Fig. 6:**
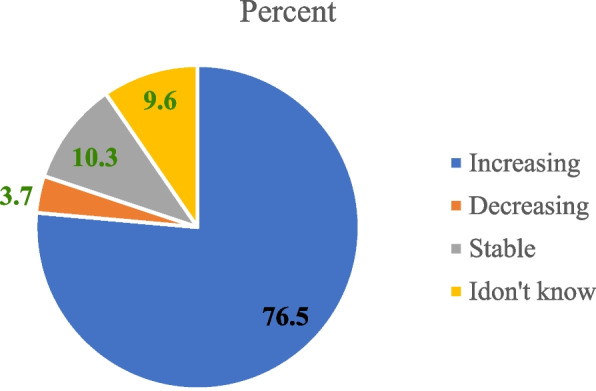
Response of respondents on intensity of crop raiding of DM in the study area

According to the respondents, compared to the olive baboon (*Papio anubis*) and grivet monkey (*Chlorocebus aethiops*), DM make relatively less conflict with the communities in the area. However, the conflict is increasing from time to time 76.5%, (*n*= 104) (Fig. 6).

**Fig. 7:**
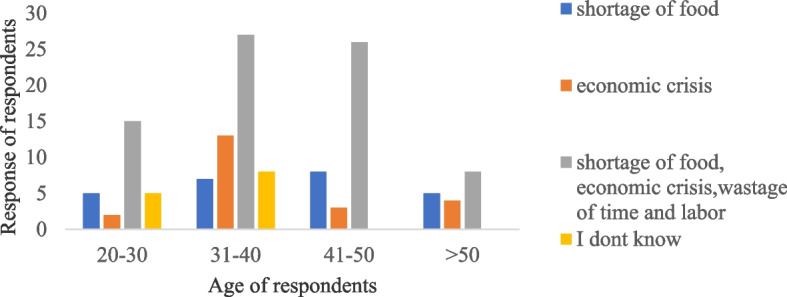
Response of respondents in different age groups about the consequence of HDMC on community members

Most of the respondents 55.9%, (*n* =76) mentioned, shortage of food, economic crisis, wastage of labor and time were the major consequence of the HDMC in the area. There was significant difference in respondents’ response among different age groups concerning the consequence of HDMC (χ^2^ = 18.448; df = 9; *p* < .0.05) (Fig. 7).

**Fig. 8:**
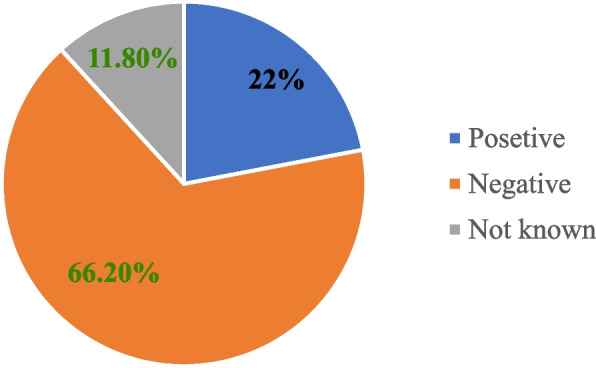
Response of respondent about their attitude towards the conservation of DM

Majority 72.1%, (*n* = 98) of the respondents believed that the number of DM is increasing in the study area; while 9.6% (*n* = 13) of them replied that they don’t know about the trend of its population. Half of the respondents 50%, (*n* = 68) estimated that a single troop of DM consists from 6 to 10 individuals while 18.4 %, (*n* = 25) of the respondents estimated from 3 to5 individuals per troop. About half 50%, (*n* = 68) of respondents have the trend of cultivating their crops near to the forest edge (< 100m) (Table 5). Majority 66.2%, (*n* = 90) of the respondents perceive DM negatively, while some 22 %, (*n* = 30) have positive attitude (Fig. 8). There was a significant difference among the responses of respondents in different educational status towards the conservation of DM (χ^2^ = 13.192; df = 6; *p* = 0.05).

**Table 5:** Response of respondents about the trend of crop raiding, population size and attitudes of the community towards DM

Factors	Responses	Frequency	Percentage
Crop raiding trend of De Brazza’s monkey	Increasing	104	76.5
	Decreasing	5	3.7
	Stable	14	10.3
	Not known	13	9.6
Current population size of De Brazza’s monkey	Increasing	98	72.1
	Decreasing	11	8.1
	Stable	14	10.3
	I don’t know	13	9.6
Number of De Brazza’s monkey in a single troop	three -five	25	18.4
	six -ten	68	50
	eleven-fifteen	23	16.9
	sixteen -twenty	7	5.1
	It is difficult to know	13	9.6
Attitudes of the community towards De Brazza’s monkey	Positive	30	22.1
	Negative	90	66.2
	Not known	16	11.8

In the present study area, 86.7%, (*n* = 118) of the respondents stated that they use dogs, scarecrows, chasing, and guarding to keep problematic animals away from their crops. Among these 44.9%, (*n* = 61) of them consider guarding as the most effective method followed by chasing, 22.9%, (*n* = 31), and scarecrow 8.8%, (*n* = 12). Majority 90.4%, (*n* = 123) of respondents indicated that there is no any compensation to the damage caused by the problematic animals. In addition, 45.6% (*n* = 62) of the respondents, replied that they asked the government and other concerned stockholders for permission to kill problematic animals.

### Discussions

The majority of participants in the current study indicated the presence of HWC and damage of agricultural crops in the study area, primarily caused by NHP. Similar studies conducted in Uganda [29], Zambia [30], and Ethiopia [31–34] revealed that primates are the most commonly identified crop raiding animals in their research areas.

In the study area, habitat degradation, an increase in the population size of DM, and human proximity to the forest edge were the main causes of HDMC This result is consistent with those of [35] in the Gera District, [36] in and around Borena Sayint National Park, [32] in Dega Damot District and [31] in the Ethiopian highlands, which all identified human closeness to the forest and habitat disturbance as the main drivers of HWC. During field observations, it was seen that the research area's dense forest was destroyed by urbanization, new settlements, and the expansion of agricultural land and coffee plantations.

The majority of respondents stated that, though DM was not considered a pest primate before 20 years, it is currently considered one of their village's pest primates. The monkeys' unusual behavior may have been brought on by human actions that destroyed their habitat for many reasons. According to research done in Uganda and Kenya by [15], the growth of subsistence farms in the Tororo District has put strain on DMs and baboons. As a result, both baboons and De Brazza’s monkeys have been accused of crop-raiding and are now being persecuted. The study also demonstrated the impact of human activity, which drove DM to other areas of Kenya's tiny forests and put it under a lot of strain [37] described as crop raiding was reportedly more difficult in places with significant levels of deforestation. Because natural food sources are diminished in these areas, local people may chase and kill DMs to stop them from crop raiding.

The majority of people in KBR are unfamiliar with DM. This result is consistent with the findings of a primate survey report published in Kenya by [38], which reported that, with the exception of a small group of Samburu people known as the "Ndorobo," who depend on honey, fruits, and herbs from the forest for their subsistence, most local residents of Mathews Forest are unaware of DM and have never seen it. Similarly, people who live on the periphery of the KBR and make their living from gathering fruits, spices, firewood, charcoal, and forest coffee plantations as well as traditional honey production are the local people who are familiar with DM in the current study.

According to the finding of the current study, DM raid fields regularly between the hours of 6:00 and 7:00 AM and 11:30 and 12:30 PM. This could be the result of its bashful demeanor and fear of being noticed by farmers. Nonetheless, the research done by [39] in the Midre-Kebid Abo Monastery Gurage Zone revealed that NHP tended to favor the daytime. Thus, the results of this study and the current study's outcome are at odds. The results of the current study are consistent with those of [38] in Kenya, which described DM as being more cautious and escaping in regions that humans frequent.

In this study, severe crop damages by DM were seen in the wet season (June-August) and dry season (December - February). This may be related to the fact that some crops, such maize, mature from June to August and that coffee and barley mature from December to February. It's also possible that there could be shortage of naturally occurring food sources, primarily fruits, during these months. The results of this study support those of [29], who said that baboon damages to maize crops occur most frequently in June in Uganda.

The result of the current study revealed maize as the most raided crop by DM followed by coffee. Different reasons can be given why maize and coffee are favored by DM. In case of maize the reason might be its sweetness and ease to be handled and takeaway from the farm to forest. Concerning coffee, it is cultivated under the shade of trees which might be fortunate for the monkey to visit at any favorable time and its fruit is also sweet when it ripens. This result is in lines with the finding of [39] who reported maize as the most vulnerable crop to crop raiders.

NHP and other problematic animals can cause yield losses of different crops. In the study area respondents reported crop yield loss of 20-30 % in a single cropping season by DM. During focus group discussion, the group discussants raised that the damage has created shortage of food and economic crisis in addition to wastage of time and labor. This finding is comparable with the finding of [20] who reported that most of the households lose 10 to 25% of their crops to wild herbivores in and around Alitash National Park in north Ethiopia, However, the yield loss in the current study is smaller than [21] who reported 85.9% crop yield loss by gelada baboon in and around the Semen Mountains National Park. In addition, majority of the respondents revealed that the intensity of crop raiding DM is increasing from time to time. Similarly increased in the intensity of crop damage by wildlife species was reported by [37] in and around Midre-Kebid Abo Monastery in Gurage Zone, [22] Zegie Penisula in Lake Tana, [20] in and around Alitash National Park.

The result of the study showed that most local residents of the study area perform farming activities close to forest edge as close as less than 100 meters. This can create interaction between humans and De Brazza’s monkey that could lead to HDMC. This result is similar to the finding of [11, 36] who reported that conversion of primate habitats into agricultural land creates the potential for conflict between hungry primates and local people. This outcome is consistent with [36]'s observation that the documented conversion of primate habitats into agricultural land raises the possibility of confrontation between the local population and famished primates in and around Borena Saint National Park.

The majority of respondents whose farmland is closer to forest edge have negative attitude towards the conservation of DM due to its crop raiding behavior and about five De Brazza’s monkeys were killed at Keja and Araba villages of the present study area during the time of data collection in revenge to crop raiding. This finding is consistent with the result of research done in the Ethiopian Highlands by [31], in Dega Damot West Gojam by [40], and in Zegie Penisula [34], and around the vicinity of the Wof-Washa Forests in North Shewa [41]; who observed that the local people have negative attitude towards NHP like grivet monkeys and problematic animals, because of their crop raiding behavior and killing of domestic animals. Similarly, [42] in Zimbabwe, reported that the local farmers had a negative attitude toward baboons.

The present study revealed that the majority of respondents use guarding as the main method to reduce crop damage by NHP. According to the information gathered from focus group discussions; protecting DM is somewhat difficult compared to other NHP because of its shy and cryptic behavior and concealing itself from human observation and due to its time of crop raiding; which is early in the morning, before farmers wake up and in evening after farmers returned to home. Similarly, guarding was reported as the main method of protecting crops from problematic animals by [41] around Wof-Washa Forest, Nort Shewa, [43] around Yegof National Forest priority Area, South Wollo, [40] Dega Damot west Gojjam, [39] Midre-Kebid Abo Monastery Gurage Zone, and [36] in and around Borena Sayint National Park [44] in Kenya, [29] in Uganda, and [42] in Zimbabwe also stated that the majority of the local people in their research areas use guarding to keep NHP out of their agriculture areas.

## Conclusion

According to the result of current study, the root causes of HDMC conflict were habitat destruction, human proximity to the forest edge and increasing of DM population. The HDMC in the current study area was due to crop damage and damage of beehives; which led the local people food insecurity and economic crisis. Farmers use different techniques in order to minimize crop damage. However, these methods require additional labor force and are time consuming. Consequently, most of the local communities had negative attitude towards the conservation of De Berra’s monkey. HDMC is increasing from time to time and in the absence of appropriate management plan, the problem will get worse in the future and could lead local extinction of the species. Therefore, giving appropriate attention is crucial to resolve the existing problem. Farmers also need to be encouraged to shift their crop productions to an unpalatable crop by DM. More awareness creation among local communities on the importance of wildlife and forest is necessary to mitigate the pressure of local people on wildlife and the forest. The local people who live close to the forest edge and depend on it for their livelihood should be taken into consideration by government officials, non-governmental organizations, and interested parties. These people should be helped by various employment alternatives that can reduce their reliance on natural resources in order to preserve the forest and wildlife.

### Supplementary Information

**Supplementary Material 1.** 

## Data Availability

The data used and analyzed during the current study are available from the corresponding authors upon request.

## Abbreviations

DM: De Brazza’s monkey
EWCA: Ethiopian Wildlife Conservation Authority
HDMC: Human - De Brazza’s Monkey conflict
HWC: Human-wildlife conflict
KBR: Kafa Biosphere Reserve
NABU: Nature and Biodiversity Conservation Union
NHP: Non-Human Primates
