# Supplementary material for: Human-De Brazza’s monkey conflict in Kafa Biosphere Reserve, Kafa Zone South West, Ethiopia
Source: BMC Zool. 2024 Aug 12;9:20. doi: 10.1186/s40850-024-00210-2 (PMC11318138; doi:10.1186/s40850-024-00210-2)
Supplement: Supplementary file 1 — Supplementary Material 1. [file 40850_2024_210_MOESM1_ESM.docx]

**Appendices I: Images related to the results of the study.**


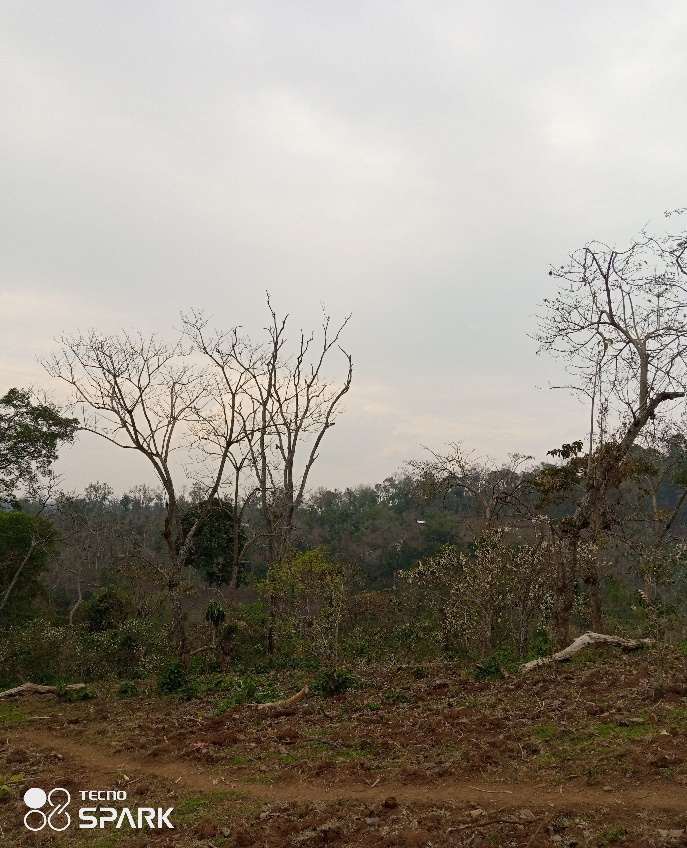

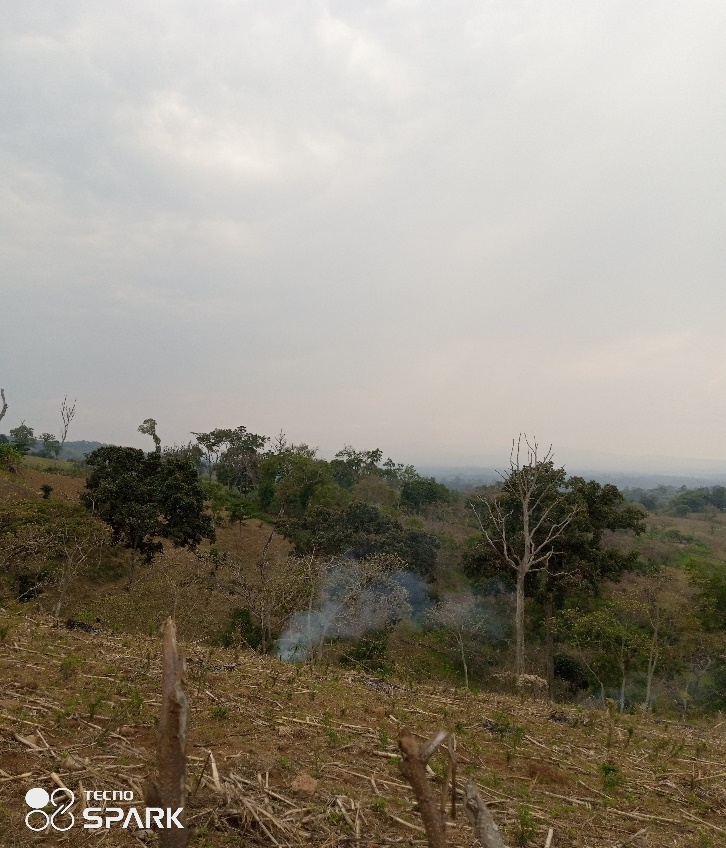


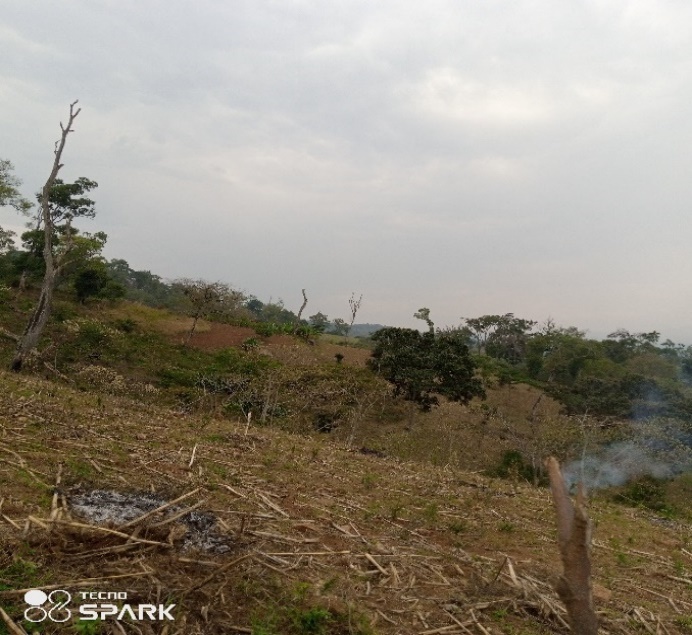

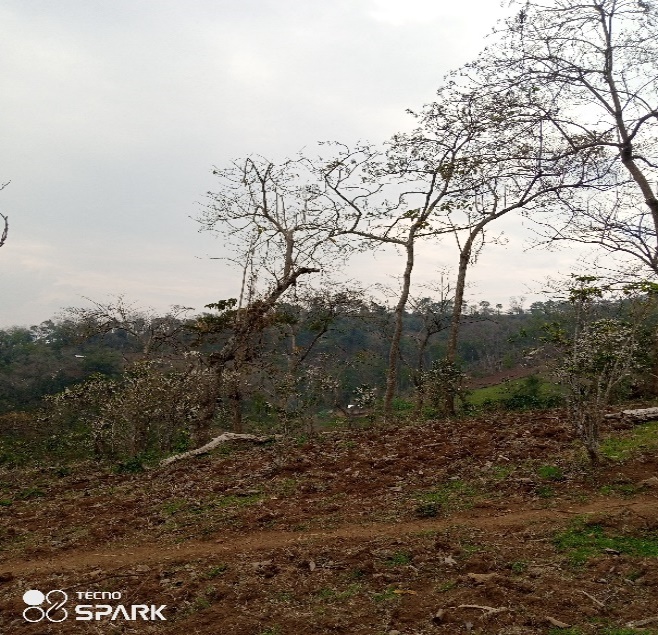


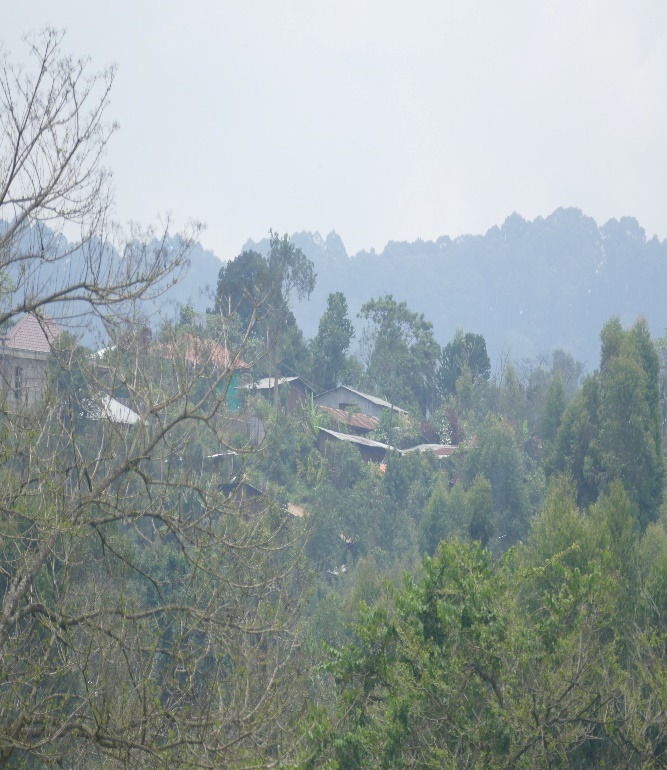

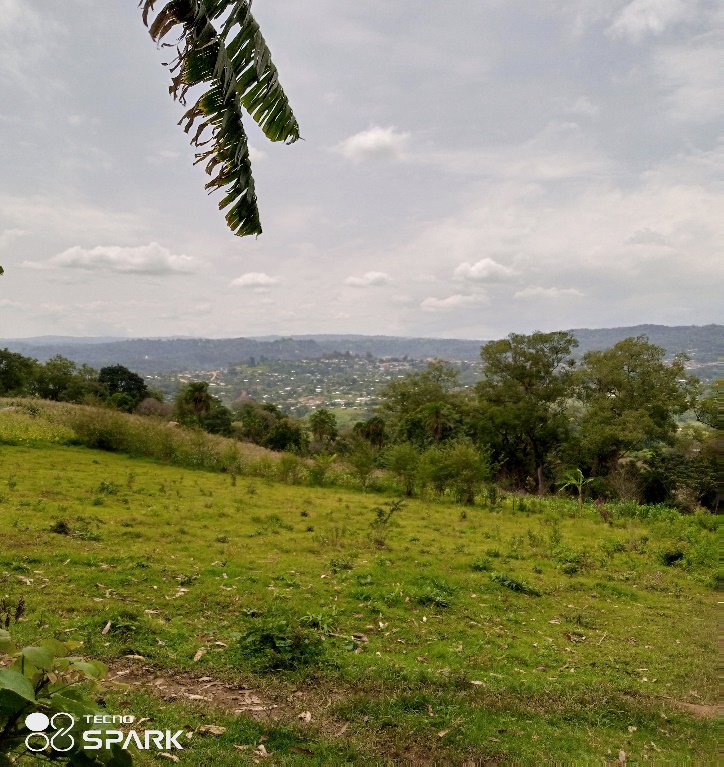


Damage of forest for different purposes.


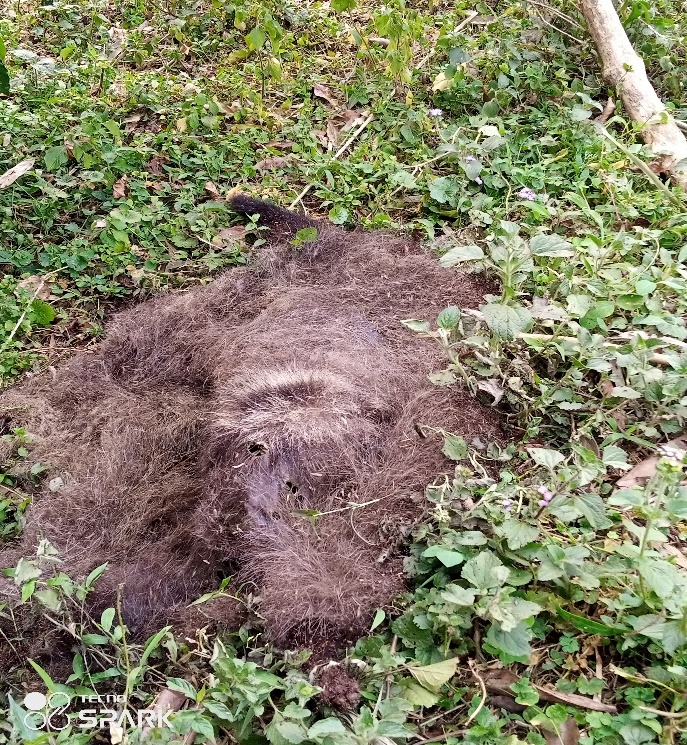

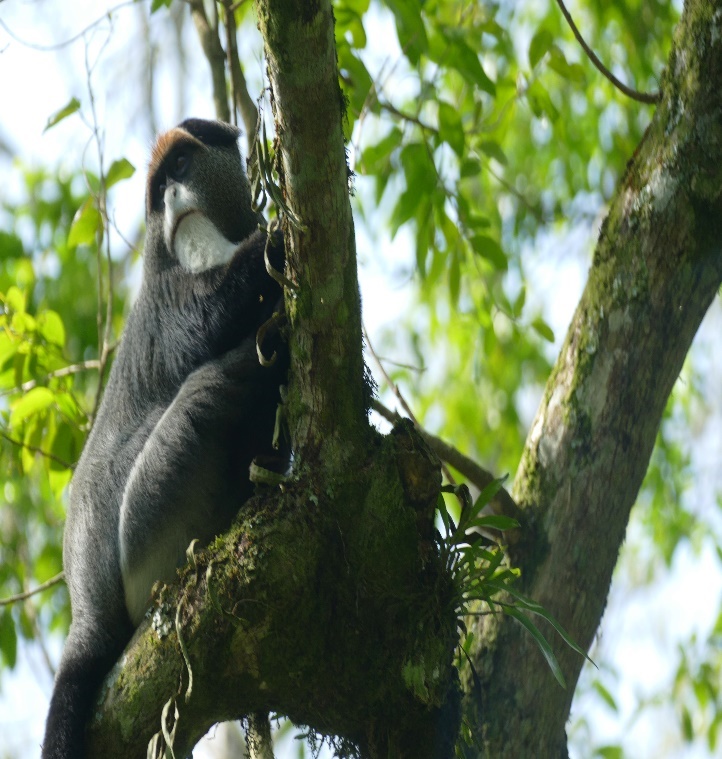


De Brazza’s monkey killed by farmers Alive De Brazza’s monkey.


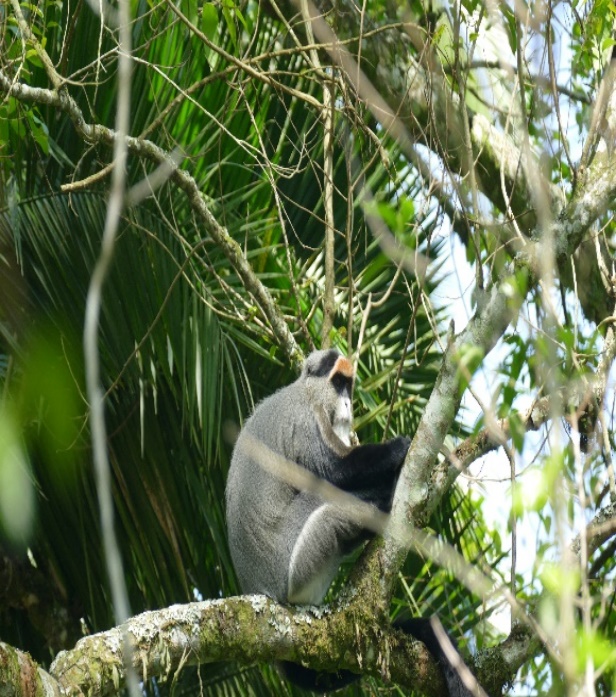

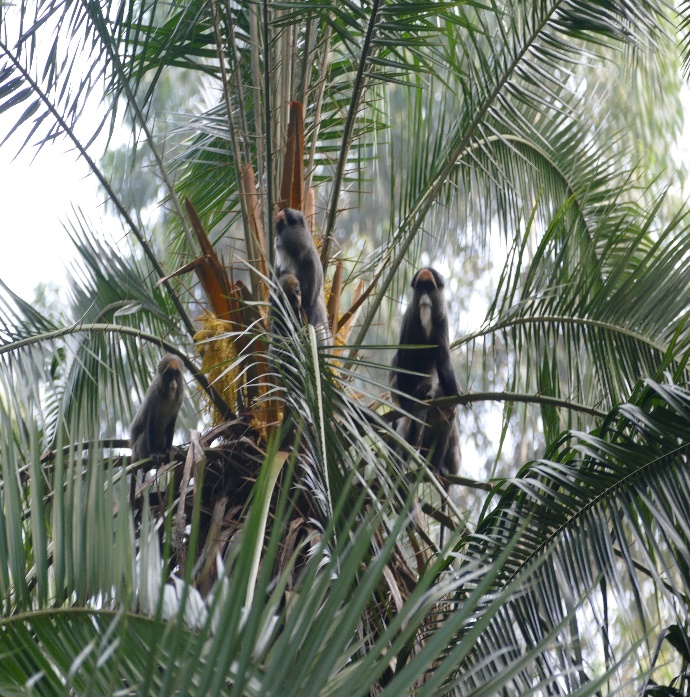


De Brazza’s monkey in the forest


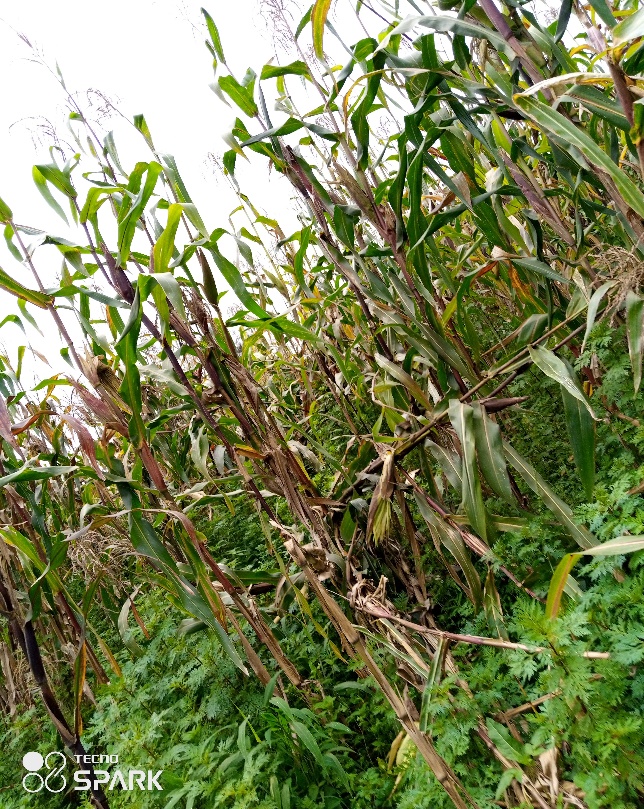

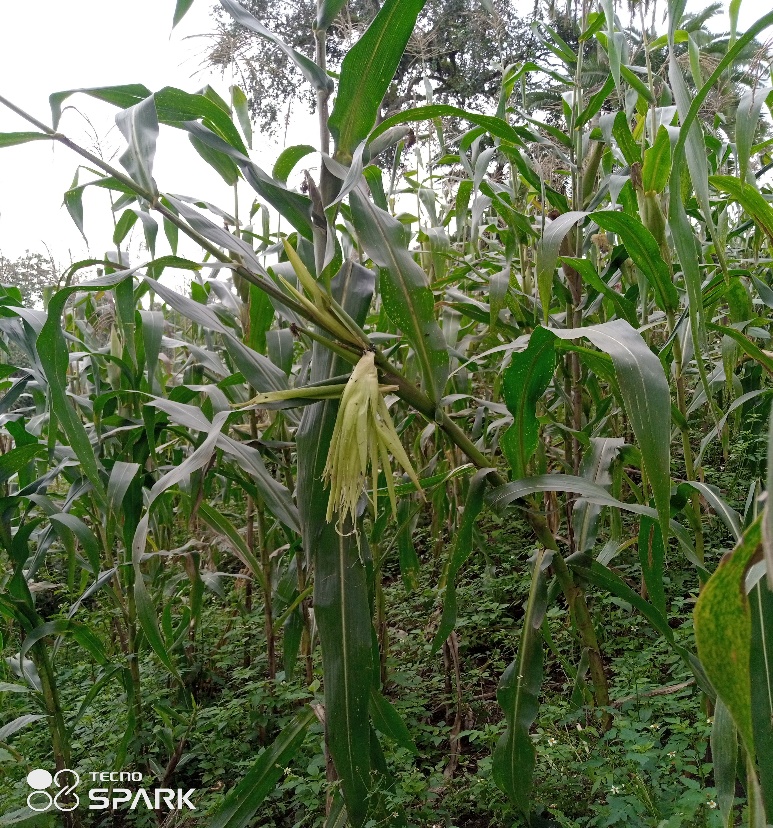


Maize farm Raided by De Brazza’s monkey.

**Appendices II: Questionnaire**

**Questionnaire**

**House hold survey**

**Questionnaire for the title “Assessment of Human De Brazza’s monkey Conflict in Kafa  Biosphere Reserve, Kafa Zone south west, Ethiopia”.**

**I. Direction.**

**Dear respondent**: I am requesting you to respond for the questionnaire provided bellow for the purpose of research. I assure that your answers will be kept confidentially about the confidentiality of your responses before, during and after the research. I assure that your secrecy and the information obtained from you will only be used specifically for the study in question. You can withdraw from the questionnaire and discussion at any time.

**** underline your answer from the given choices below****

Best Regards.

**II. General information**

Region ______ Zone ______ Woreda _________ Kebele _______ Village _________

**III. House hold characteristics**

1. Code of the Respondent________________ **2.** Sex ____ **3.** Age________

4. Marital status:

**1.** Married **2.** Single

**3.** Divorced 4. Widowed

**4.** Education level of household head:

**1**. Illiterate **2**. Primary education (1-4) **3**. Junior (5-8)

**4**. Secondary education (9-10) **5.** College & above

5. Landholding (ha):

**1.** <1ha **2.** 1 ha **3**. 1.5-2.4ha

**4.** 2.5-3ha **5.** >3ha

6. How long you lived in this village?

**1.** < 10yrs **2.** 10-20yrs **3.**21-30 yrs.

**4.** 31-40 yrs. **5.** 41-50 yrs. **6.** >50yrs.

7. From what sources do you get money / income?

**1.** From crop production and animal rearing **2.** From crop production, animal rearing and trade

**3.** From crop production, animal rearing and others **4.** From crop production and trade

**5.** From crop production and others **6.** From crop production, trade and others

**7**. From crop production, animal rearing, trade and others

8.What type of crops do you cultivate? (Please put << **x**>> in the box for your answer).

| S/No | Type crop | Yes | No |
| --- | --- | --- | --- |
| 1 | Do you produce maize |  |  |
| 2 | Do you produce Barley? |  |  |
| 3 | Do you produce Bean? |  |  |
| 4 | Do you produce Pea? |  |  |
| 5 | Do you produce Haricot bean? |  |  |
| 6 | Do you produce Sorghum? |  |  |
| 7 | Do you produce Teff? |  |  |
| 8 | Do you produce Vegetable? |  |  |
| 9 | Do you produce Fruits? |  |  |
| 10 | Do you produce Coffee? |  |  |

9. Which animal/s do you rear? (Please put << **x**>> in the box for your answer).

| S/No | Type of animal | Yes | No |
| --- | --- | --- | --- |
| 1 | Do you rear Cattle? |  |  |
| 2 | Do you rear Sheep? |  |  |
| 3 | Do you rear Goat? |  |  |
| 4 | Do you rear Hen? |  |  |
| 5 | Do you rear Equine? |  |  |

10. Do you have a private grazing land?

**1**.Yes **2.** No

**V. Human-De Brazza’s Monkey Conflict**

11.Is there problem of crop damaging in your village?

**1.**Yes **2.** No

12.What are the factors those damage crops in your village?

**1.** Pest animals **2.** Insects

**3.** Disease **4.** Climate change **5.** I don’t know

13. Do you know the De Brazza’s Monkey?

**1.**Yes **2.** No

14.Do other peoples in your village/community know De Brazza’s Monkey?

**1.** All of them know it **2.** Most of them know it

**3.** Some of them know it **4.** Most of the don’t know it

15.Is there any conflict between human and De Brazza’s Monkey?

**1.** Yes **2.** No

16.If there is conflict between human and De Brazza’s Monkey, what is the cause?

**1.** Due to habitat destruction **2.** Due to population increase

**3.** Due to preference of farm crops **4.** Proximity of farm land to the forest **5.** I don’t know

17. How long does the De Brazza’s Monkey started crop raiding behavior?

**1.** It is pest in nature **2.** About 20yrs

**3.** 10-20 yrs. **4.** < 10yrs **5.** I don’t know

18. The impact of the conflict is?

**1.** Crop raiding **2.** Crop raiding and Damage of Beehive

**3.** Animal predation **4.** Human attack **5.** I don’t know

19. In which season crop raiding status becomes serious? Which type of crops were raided at each season? ______________________________________________________________________.

1.Summer 2. autumn 3. winter 4. Spring

20. In which months were crop raiding was sever in your village?

**1.** September to November **2.** December -February

**3.** March - May **4.** June to August **5.** I don’t know

21. At which time of the day De Brazza’s Monkey frequently raids crop?

**1.** Early in the morning (6:00-7:00 AM) **2.** Late morning (8:00-10:00 AM**)**

**3.** Midday (11:00-12:00 AM) **4.** After noon (1:00-3:00 PM)

**5.** Early evening (4:00-5:00 PM) **6.** Late Evening (11:30-12:30 PM) 7. I don’t

22. What type of crops it raids most of the time? (Please put << **x**>> in the box for your answer).

| **S/No** | **Type of crops** | **Yes** | **No** | **I don’t know** |
| --- | --- | --- | --- | --- |
| 1 | Does De Brazza’s Monkey raids maize |  |  |  |
| 2 | Does De Brazza’s Monkey raids |  |  |  |
| 3 | Does De Brazza’s Monkey raids |  |  |  |
| 4 | Does De Brazza’s Monkey raids |  |  |  |
| 5 | Does De Brazza’s Monkey raids |  |  |  |
| 6 | Does De Brazza’s Monkey raids |  |  |  |

23. How much percent of your crop was damaged by De Brazza’s Monkey in a single farming season? **1.<**10% **2**.10-20% **3.** 21-30%

**4.**31-40% **5.** >40% **6.** I don’t know

24. How about the recent crop raiding trend of De Brazza’s Monkey in your village?

**1.** Increasing **2.** Decreasing

**3.** Stable **4.** It is not known

25. What about the current population status of De Brazza’s Monkey around your village?

**1.** Increasing **2.** Decreasing

**3.** Stable **4.** It is not known

26. Coud you estimate the population number of a single group?

**1.** 3-5 **2.** 6-10 **3.** 11-15

**4.** 16-20 **5.** >20 **6.** It is difficult to know.

27. How far is your farm from forest edge?

**1.** <100m. **2.**101-200m **3.** >200m

28. What about your attitude towards the conservation of De Brazza’s Monkey?

**1.** Positive **2.** Negative **3.** Not known

29. Do you protect your crop from damage of De Brazza’s Monkey?

**1.** Yes **2.** No

30. Which method/s do you use to protect your crop from the damage of De Brazza’s Monkey?

**1.** Guarding **2.** Chasing **3.** Using dogs

**4.** Scarecrow **5.** I don’t use

31. Which method/s is/are most effective?

**1.** Guarding **2.** Chasing **3.** Using dogs

**4.** Scarecrow **5.** I don’t use

32. Is there any support from Government or NGOs regarding the damage cause by De Brazza’s Monkey?

**1.**Yes **2.** No

33. What is your expectation from government about De Brazza’s Monkey and other crop raiding animals.

**1.** Allow to kill **2.** provide Insurance

**3.** protect forest from destruction **4.** I don’t know
